# Vista of the Future: Novel Immunotherapy Based on the Human V-Set Immunoregulatory Receptor for Digestive System Tumors

**DOI:** 10.3390/ijms24129945

**Published:** 2023-06-09

**Authors:** Paulina Chmiel, Katarzyna Gęca, Adam Michalski, Martyna Kłosińska, Agnieszka Kaczyńska, Wojciech P. Polkowski, Zuzanna Pelc, Magdalena Skórzewska

**Affiliations:** Department of Surgical Oncology, Medical University of Lublin, Radziwiłłowska 13 St., 20-080 Lublin, Polandwojciech.polkowski@umlub.pl (W.P.P.); magdalena.skorzewska@umlub.pl (M.S.)

**Keywords:** VISTA, VSIR, PD-1H, Dies1, tumor microenvironment, immunotherapy, gastrointestinal cancer, immune checkpoint

## Abstract

While gastrointestinal tumors remain a multifactorial and prevalent group of malignancies commonly treated surgically in combination with chemotherapy and radiotherapy, advancements regarding immunotherapeutic approaches continue to occur. Entering a new era of immunotherapy focused on overcoming resistance to preceding therapies caused the emergence of new therapeutic strategies. A promising solution surfaces with a V-domain Ig suppressor of T-cell activation (VISTA), a negative regulator of a T-cell function expressed in hematopoietic cells. Due to VISTA’s ability to act as both a ligand and a receptor, several therapeutic approaches can be potentially developed. A broad expression of VISTA was discovered on various tumor-growth-controlling cells, which proved to increase in specific tumor microenvironment (TME) conditions, thus serving as a rationale behind the development of new VISTA-targeting. Nevertheless, VISTA’s ligands and signaling pathways are still not fully understood. The uncertain results of clinical trials suggest the need for future examining inhibitor agents for VISTA and implicating a double immunotherapeutic blockade. However, more research is needed before the breakthrough can be achieved. This review discusses perspectives and novel approaches presented in the current literature. Based on the results of the ongoing studies, VISTA might be considered a potential target in combined therapy, especially for treating gastrointestinal malignancies.

## 1. Introduction

As has been established, the organism’s response to the appearance of a neoplastic process is based on numerous complex immune mechanisms, which have been successfully targeted in anti-cancer therapy for many years [1]. A key role in controlling these mechanisms is played by immune checkpoints, which are physiologically responsible for maintaining self-tolerance and limiting tissue damage occurring during an anti-microbial immune response [2]. However, immune checkpoints in the microenvironment of various tumors serve a different role. Their expression is associated with decreased activity of T cells as well as decreased anti-cancer response. Consequently, overexpression of the immune checkpoint becomes a factor often associated with a worse prognosis, leading to reduced response rate (RR) and overall survival (OS) [3]. As such, the appliance of immune checkpoint inhibitors stimulates the upregulation of neoplasm control mechanisms [4,5]. Recent years yielded evidence of the benefits of further development of immunotherapy based on B7 family members, such as V-domain Ig suppressor of T-cell activation (VISTA). Considering the possibilities of broad applicability, good tolerance, and promising research results, this approach could be evaluated as a treatment option for various solid tumors [6].

The prime examples of malignancies that may be treated with VISTA inhibitors are gastrointestinal tumors (GITs), especially considering the expression of this molecule in various GITs [7,8]. They are a heterogeneous group consisting of esophageal, gastric, pancreatic, hepatobiliary, and colorectal cancer [9]. They all differ in their specific microenvironments, tumor-induced immune responses, and currently available treatment options [10]. It is imperative to find new therapeutic approaches capable of targeting all GITs, regardless of their wide range and variety [11]. To date, only a few immunotherapies have been recommended to treat GITs in contrast to other malignancies, such as urothelial cancer [12]. Pembrolizumab, a programmed cell death protein (PD-1) inhibitor, is one of the approved immunotherapies and has been successfully used in the therapy of patients diagnosed with pre-treated unresectable solid tumor with microsatellite instability (MSI-H) or mismatch repair deficiency (dMMR), due to its rich T-cell infiltration [13,14]. The KEYNOTE-811 clinical trial presented the efficacy of pembrolizumab combined with trastuzumab, an HER-2 inhibitor, in the treatment of advanced HER-2-positive gastric cancer. The trial showed that adding pembrolizumab to trastuzumab and chemotherapy markedly reduces tumor size, induces complete responses in some participants, and significantly improves the objective response rate [15]. Additionally, the combination of pembrolizumab with fluoropyrimidine in addition to platinum-based chemotherapy was proved to be a successful treatment of advanced esophageal cancer during the KEYNOTE-590 clinical trial [16]. Based on the CheckMate-040 clinical trial, the immunotherapy of hepatocellular carcinoma has been approved as a second-line treatment using nivolumab, a PD-1 inhibitor [17]. However, pancreatic cancer still lacks approved immunotherapy that would provide satisfactory effectiveness [18]. Lack of compromise on the immunotherapeutic approaches in GITs promotes the search for novel treatment strategies, especially considering the significant impact of TME and microbiota in tumorigenesis observed in these malignancies.

Even though the development of immunotherapy has accelerated in recent years, therapy resistance or intolerance continues to occur, leading to suboptimal clinical response. In general, approximately only 30–40% of solid tumors respond to the treatment with a single PD-1/PD-L1 blockade, proving significant resistance to these agents used in monotherapy [19,20]. The search for effective therapeutic combinations and the identification of new therapeutic targets remains one of the main issues with the current immune oncology. This paper aims to explore other possibilities regarding the treatment of GITs, focusing on the inhibition of VISTA-a potential breakthrough in managing this type of tumor.

## 2. VISTA: An Overview

VISTA is a V-domain Ig suppressor of T-cell activation, also known as SISP1, VISTA, C10orf54, B7H5, PD-1H, Dies1, Gi24, and DD1α, VSIR, and human V-set immunoregulatory receptor, among others [6]. The Vsir gene located on chromosome 10 in mice stands for type I transmembrane protein with 309 amino acids (aa) [21]. The extracellular domain of VISTA is composed of a single IgV domain with 136-aa, linked to a 23-aa stalk region, a 21-transmembrane segment, and a 97-aa cytoplasmic domain that does not contain tyrosine-based activation motifs [22]. Interestingly, the exact role of the intracellular domains remains unknown [21]. An 85,6% similarity between mouse and human VISTA indicates a vital role of such conserved protein [6,22]. Indeed, human VISTA contains 311 aa with a 32-aa signal peptide, a 130-aa extracellular IgV domain, a 33-aa stalk region, a 20-aa transmembrane domain, and a 96-aa cytoplasmic tail [23]. The sequencing research linked VISTA with the B7 family, among which PD-L1 showed the closest resemblance to the VISTA considering the extracellular domain [22]. However, some studies suggest that the origin of VISTA lies in a different family. Despite apparent similarities to the B7 family, VISTA has multiple unique factors, for instance, a large IgV domain or histidine residues. Other distinctive factors are the absence of the IgC domain, additional cysteines in the IgV domain (Cys44, Cys83, Cys144), and the structure of ten beta-strands with extra H-strand and two disulfide bonds [24,25]. Moreover, it has been discovered that VISTA bears a resemblance to the CD28 family as it can serve as both a receptor and a ligand [26,27,28]. VISTA has dual immunosuppressive functions, acting as a ligand on myeloid cells and APCs where it uses PSGL-1 as its receptor and also functioning as a receptor on cytotoxic T cells, where it binds with VSIG-3 as its ligand [29].

**Figure 1:**
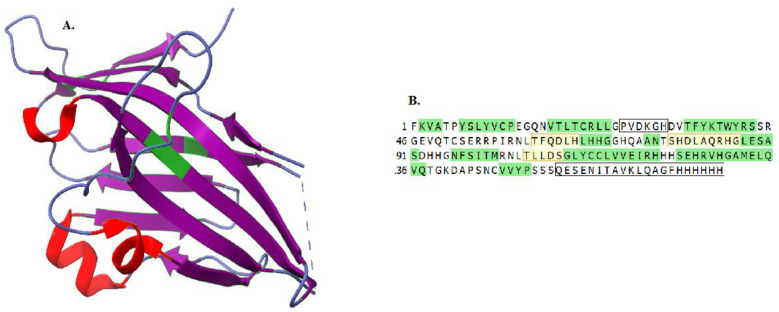
(**A**) Molecular structure of VISTA extracellular domain (ECD) with labeled secondary structure (red for alpha-helices and purple for beta-sandwiches) and cysteine molecules (depicted in green) distinct from other members of the B7 family. The extracellular organization of the VISTA molecule is crucial in binding ligands and transducing signaling; deviant structure allows VSIR to act both as ligand and receptor. (**B**) The primary amino acid sequence of human VISTA ECD with labeled secondary structure (green for beta-sandwich and yellow for alpha-helices). Boxes for no corresponding structure residue [26,27,28].

The conformation of VISTA consists of three alpha-helices in the beta-sandwich [24,29,30,31]. The key part responsible for V-set immunoregulatory receptor function seems to be its extracellular domain structure (Figure 1). Critical differences in histidine quantity in VISTA, accounting for 8,6% of the extracellular residue, seem to play a crucial role in the inhibition of T cells [32]. The mentioned difference between the B7 family and VISTA regards the orientation of histidine residues. In VISTA, they are located at a distance from the cell membrane, affecting the molecule’s extracellular organization [32]. As a result, the interaction with counter-receptors and their binding to the T cells becomes possible [32]. There are two certain binding partners for VISTA, namely, VSIG3 and PSGL-1 [30]. Although VSIG3 interacts with VISTA in vitro, its physiological role is not yet fully understood and requires further research, especially as a novel potential VSIG3-VISTA pathway in immunotherapy [33]. In addition, an acidic environment, the characteristic of malignancies, was proven to increase the binding strength of PSGL-1, potentially opening another way of inhibiting this pathway [34]. Novel studies showed that galectin-9 is a possible ligand for VISTA and can affect the apoptosis of T cells [35]. Through VISTA, galectin-9 can induce leakage of the proteolytic enzyme granzyme B from the intracellular granules of cytotoxic T cells and lead to their programmed death [36]. The consequence of these factors is the creation of a pro-cancerous environment that can facilitate the initiation and progression of cancer.

Although the currently available data are insufficient and, thus, are unable to elaborate on this subject further, several differences in the VISTA expression in mice and humans have been discovered. Significantly, murine VISTA expression is largely restricted to hematopoietic cells with predominance to macrophages, myeloid dendritic cells (DCs), and CD4+ T lymphocytes (19). However, in humans, this protein is highly expressed on the surface of monocytes, lymphoid CD11cloCD123+HLA-DR+, myeloid CD11c+CD123loHLA-DR+ DCs, at a moderate level on CD4+ and CD8+ T cells, and at a low level on CD56loNK cells but not on CD56hi NK cells [23,37]. In nonmalignant conditions, VISTA is expressed mainly on hematopoietic tissues, such as bone marrow, spleen, and thymus, or tissues with rich leukocyte infiltration, such as lungs or intestines [23]. The expression was also found in the placenta [23].

Additionally, some cells showed an abundant VISTA expression, as it was found in microglia [38,39]. Another factor differentiating VISTA from other members of the B7 family is the microenvironment-dependent expression of this protein. Studies show that the expression is also dependent on inflammation and correlates with the activation of the immune system in the TME. After a brief in vitro culture with α-CD3 or IFN-γ and LPS (lipopolysaccharide) as activators, the expression of VISTAs on both T cells and myeloid cells disappears, regardless of the presence of the stimulant [22]. Furthermore, the expression of CD4 T cells in vivo is correlated with their activation status and cytokine microenvironment during an active immune response [22]. The expression of VISTA also varies depending on inflammatory conditions present in other diseases, as the overexpression can be initiated by IL-10, Toll-like receptors (TLR), IFN-γ, or foreign and self-antigens [37,40]. In malignancy, VISTA was found in various tumors, such as gastric cancer, melanoma, leukemia, and bladder cancer. It was mainly expressed on the cells of the tumor microenvironment (TMEs) and tumor-infiltrating leukocytes (TILs) rather than on tumor cells themselves [7,28,41,42,43,44]. Interestingly, VISTA expression in tumors can be dependent on methionine metabolism. The compounds derived from methionine metabolism promoted the translation of immune checkpoints, including PD-L1 and V-domain Ig suppressor of T-cell activation (VISTA), in tumor cells. In mouse models, tumor size was reduced by dietary methionine reduction [45]. Among the wide range of human malignancies, the most significant expression of tumor cells was found in mesotheliomas, gastric, and hepatocellular cancers [41,46,47,48]. The broad expression of VISTA in TMEs seems to be a promising target in tumor therapy. The study on gastric cancer by Boger et al. presented evidence that the expression of VISTA was correlated with multiple factors: tumor localization; Lauren histological phenotype; Epstein–Barr virus infection; KRAS- and PIK3CA-mutational status; and PD-L1 expression [41]. Zhang et al. showed that in hepatocellular cancer, the expression level of VISTA on tumor cells, but not in TMEs, correlated with a better survival rate [48]. Furthermore, relevant studies have proved that immunotherapy-resistant pancreatic cancer manifests with a high VISTA expression on tumor-associated macrophages [49]. Each of the studies mentioned above suggests that using VISTA inhibitors in polytherapy might be beneficial to the outcome of the anticancer (Table 1) [43,50,51].

## 3. VISTA: Immunological Functions and Their Role in Malignancies

TME in various malignancies is created by multiple interacting cells. Both tumor cells and immune system cells are responsible for mechanisms of tumor tolerance or suppression [54]. The trend of anti-tumor immunity can be shaped depending on the overall process. Although functioning immune systems, tumors eventually evade immune surveillance by shaping an immunosuppressive microenvironment. Despite the diversity of neoplastic mechanisms, TME cells can be divided into two main groups, anti-tumor, and tumor-promoting [55].

Anti-tumor cells are mainly effector T cells, including cytotoxic CD8+ T cells and effector CD4+ T cells, natural killer cells (NK), dendritic cells (DC), and macrophages. Macrophages can be divided into two groups depending on their polarization, M1 macrophages have anti-tumor properties, and M2 macrophages are counted as pro-tumor cells [56]. It is noteworthy that the expression of VISTA can be induced by M2 macrophages, particularly through the involvement of histamine and its receptors [57]. The tumor-promoting cells are Tregs, MDSCs, and others, such as neutrophils and natural killer T Type 2 cells (NKT2) [58]. T cells are the primary agents responsible for anti-tumor immune response, executing it by death ligand-induced apoptosis or secretion of IFN-γ and TNF-α to induce tumor cells’ cytotoxicity. Moreover, CD4+ T cells are actively involved in controlling and promoting DCs and CD8+ T-cell proliferation [59]. DCs, as the most specific antigen-presenting cells (APCs), generate back the costimulatory signals with CD80/CD86 that increase the T-cell activation. Furthermore, DCs produce proinflammatory cytokines TNF-α, IL-6, IL-8, and IL-12 [60]. At a particular stage of tumor development, loss of MHC I becomes characteristic, which may result in decreased immune response, yet NKs, through the mechanism of “missing self”, are still capable of eliminating tumor cells [61].

Essential tumor-promoting cells are Tregs and MDSCs. They can interplay with each other, affect anti-tumor cells, and coexist with tumor cells. Tregs are responsible for immune homeostasis and tolerance, but in TME, they create immunosuppressive conditions in several mechanisms [62]. They produce immunosuppressive cytokines, such as TGF-β, IL-10, and IL-35. Moreover, Tregs inhibit CD8+ T cells by TGF-β and the generation of memory CD8+ T cells through CTLA-4 [63,64]. Tregs inhibit NK cell proliferation, IFN-γ production, and cytotoxicity. CTLA-4 can bind to CD80/CD86 on the DC to down-regulate the costimulatory signal [65]. As mentioned above, Tregs support the effects of MDSCs.

**Figure 2:**
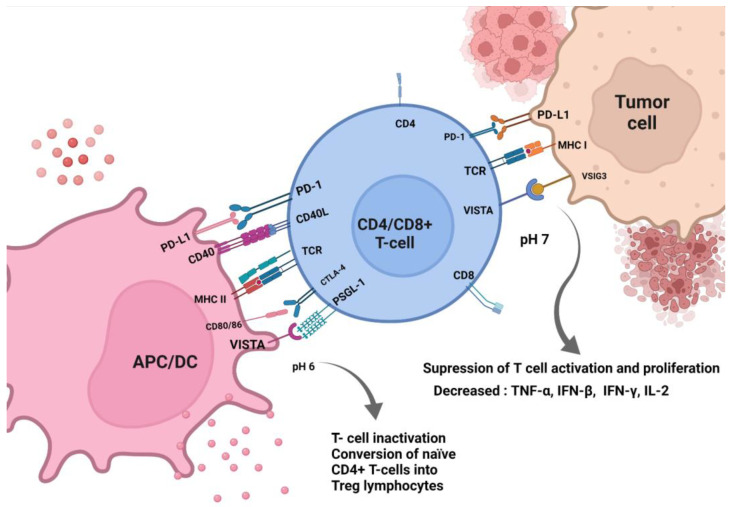
VISTA role in the TME. VISTA functions both as a ligand and a receptor. PD-1H can be expressed on TILs and MDSCs and, less commonly, on tumor cells [44,53]. VISTA binds PSGL-1 at acidic pH and VSIG3 at neutral pH, and both pathway effects are prevalent in the immunosuppression in TME [42]. By binding to its ligands, VISTA decreases the levels of proinflammatory cytokines, IL-2, TNF-α, and IFN-γ, simultaneously increasing anti-inflammatory cytokines and mediators, such as IL-10 [19]. Binding VSIG3 may result in the inactivation and suppression of the proliferation of T cells [92]. Binding to PSGL-1 in an acidic environment, which is a hallmark of TME, may cause the inhibition of T-cell receptor (TCR) signaling and cytokine production. Likewise, PSGL-1 leads to the conversion of naïve CD4+ T cells into Treg lymphocytes by inducing Foxp3 expression [93]. Considering the abovementioned mechanisms, VISTA functioning in TME results in creating favorable conditions for the tumor in the host.

VISTA’s dualistic nature seems crucial in controlling immunological reactions in both physiological conditions and cancer environments (Figure 2) [22,47,50,66]. VISTA can be a potent inhibitor for both T cells and B cells while simultaneously being an APCs stimulator. However, it is imperative to find the exact pathways responsible for regulating VISTA-mediated immunological responses, as no tyrosine-based activation motifs were found [67]. Nevertheless, there is strong evidence for the ability of VISTA to transduce the signal through the intracellular domain, which contains two protein kinase C binding sites and proline residues potentially serving as docking sites [21]. The research in mice models proved that VISTA ligand is a strong inhibitor for both naïve and memory subsets of CD4+ T cells. Significant suppression of the early activation markers: CD69, CD44, and CD62L was also observed, which indicated the blockade occurring during the early stages of the T-cell activation [22]. Furthermore, VISTA inhibited cytokine (IL-2, IFN-γ) production from CD4+ and CD8+ T cells. Importantly, no apoptosis induction has been observed in this study, and VISTA expression on tumor cells seemed to cause the induction of immune tolerance, simultaneously disrupting the protective antitumor immunity [22]. VISTA can also contribute to the conversion of naïve CD4+ T cells into Treg lymphocytes, suppressing antitumor responses [50,68].

Considering various immunological functions of VISTA, the interactions and relevant modulating factors may occur in different environments. Myeloid-derived suppressor cells (MDSC) isolated from mice infected with LP-BM5 retrovirus are proven to inhibit B-cell proliferation in vitro in a VISTA-dependent manner [69]. Le Mercier et al. showed that despite high levels of PD-L1 and lack of VISTA on tumor cells, blocking this ligand may result in TILs’ activation. Even in the absence of direct expression, VISTA seems to participate in the immune response to the proliferative process [7]. VISTA is expressed on T cells as a receptor, negatively regulating their activity. VISTA knock-out CD4+ T cells, derived from mutant PD-1H deficient mice with interference in the coding exon 1 of the Vsir gene, respond more efficiently than wild-type cells, adequately leading to the increased production of IFN-γ, TNFα, and IL-17A [25]. However, in HIV-positive patients, infected monocytes expressed higher levels of VISTA, TNFα, IL-1β, and IL6 mRNA when compared to healthy monocytes [37]. Blocking VISTA may occur in a bond reduction with VSIG3, thus causing an increase in the synthesis of CCL5, CCL3, and CXCL11, as well as IFN-γ, IL-2, and IL-17 [33]. Importantly, it has been proven that these cytokines directly stimulate anti-tumor conditions of the cancer environment, cancer progression, and the immune response to the tumor [70,71,72]. However, CCL-5, CCL3, and CXCL may have dualistic potential as they can show both protective effects and increase the neoplastic risk [73,74,75,76]. More importantly, activation of the immune system in response to PD-1H blockade without its expression suggests the existence of other alternative signaling pathways worth further research.

Furthermore, some studies suggest that VISTA can play an important role as a biomarker in gastrointestinal tumors [77]. Hou et al. demonstrated that in pancreatic cancer, VISTA expressed on tumor cells could serve as a favorable prognosis marker [78]. Thus, the expression was associated with prolonged overall survival. Zong et al. showed that the expression of tumor-infiltrating immune cells correlates with early tumor stage, MMR deficiency, and a favorable prognosis in patients with colorectal cancer [74]. Lastly, a study on colorectal cancer stated that high VISTA expression correlated with better disease-free survival (DFS), high tumor infiltrative lymphocyte, microsatellite instability, BRAF mutational status, as well as lower tumor stage [75]. Conversely, various studies have indicated that elevated expression of this molecule could be linked to an unfavorable prognosis. In melanoma, the expression of VISTA is correlated with the occurrence of ulceration, deeper Breslow thickness, lymph node involvement, and advanced stage [79]. Similarly, in non-small cell lung cancer (NSCLC), the high expression on CD4+ T cells was correlated with reduced overall survival, high rate of lymphocyte metastasis, and low secretion of cytokines, including IFN-γ, IL-2, IL-4, IL-10, and IL-17 [80]. In pancreatic neuroendocrine tumors (Pan-NETs), high VISTA expression has been correlated with unfavorable clinicopathologic features and shorter progression-free survival. However, in this study, only 25% of tumors have been marked as VISTA-high; overall expression of this molecule was confirmed in 66% of samples [81]. Furthermore, Digomann et al. showed that in pancreatic cancer, there was a significantly higher VISTA expression compared with that of a nontumorous pancreas, and it could be correlated with reduced OS [82]. When considering all the available information, it is clear that VISTA expression could function as a prognostic biomarker, with the possibility of either positive or negative implications, depending on the tumor type.

Both in vitro and in vivo research demonstrated the critical role of VISTA in controlling TME cells, affecting the therapy outcome and possible clinical approach [6]. A high expression of VISTA may result in the escape from immune surveillance and occur as a neoplastic process [83]. In several tumor models, it has been discovered that cells of TME overexpress VISTA, especially tumor-infiltrating myeloid cells, such as DCs and MDSCs. Interestingly, the exact levels of expression are found to be increased tenfold, as compared to the cells of peripheral lymph nodes [7]. In addition, VISTA-deficient melanoma tumor-bearing mice have inhibited tumor growth more effectively [84]. Recently, Hou et al. proved that in pancreatic cancer, VISTA expression on tumor cells could be correlated with prolonged overall survival, and anti-VISTA antibody treatment significantly reduced the number of metastatic nodules [85]. Additionally, in the case of pancreatic cancer, there is evidence to suggest that VISTA is associated with Toll-like receptor 4, which is known to play a critical role in innate immune responses. Blockade of these two compounds may result in a reduction in cell proliferation [86]. Similarly, Zong et al. indicated that higher expression of VISTA on immune cells could be correlated with lower clinical advancement and stage I rather than stage III of colon cancer. As mentioned before, similarly to a study on pancreatic cancer, a study on colon cancer showed longer OS in patients with high VISTA expression than those with absent VISTA [87]. Furthermore, the appliance of anti-VISTA antibodies can reverse immune suppression and modulate the tumor microenvironment with an anti-tumor effect [88]. Hypoxic and acidic TME may additionally entail not only the overexpression of VISTA on tumor-infiltrating leukocytes but also its binding to the PSGL-1 in a greater manner while inducing immunosuppression [34,89]. A poor prognosis is associated with VISTA activated by hypoxia-inducible factor 1-α (HIF-α) [90]. Le Mercier et al. proved that VISTA inhibitors significantly reduced growth in many solid tumor models (B16/OVA melanoma, B16/BL6 melanoma, MB49 bladder carcinoma, and PTEN/BRAF inducible melanoma) regardless of their immunogenic status or origin [7]. Most importantly, VISTA blockade was proven to be effective even without VISTA expression on the tumor cells [7]. VISTA overexpression was also found in patients who progressed during prior immunotherapy with anti-PD-1 or anti-CTLA-4, which can indicate immune resistance and is essentially what led us to combine therapy with many B7 family inhibitors for better results [42,91].

## 4. Mechanisms of Resistance to Immunotherapy in GI and Their Correlation with VISTA

Multiple studies present a correlation between the VISTA expression, ligand binding, and the acidic pH of the TME, especially with the PSGL-1 ligand [33,34,94]. Significantly increased activity of VISTA in lower pH may affect CD4+ T-cell inhibition and promote T-cell exhaustion, leading to a poor prognosis and a suboptimal answer to treatment in patients with an inflammatory subtype of malignancy [95,96]. A decreased extracellular pH is characteristic of solid tumors and corresponds with tumor progression, metastases, and pathology of TME [97,98]. At the same time, the hallmark of solid tumors is hypoxia, and significant pro-angiogenic activity is caused by it, with a higher level of HIFα in the tumor microenvironment. The increased levels of HIFα in TME are correlated with the immune resistance from B7 family members [90,99]. This study showed that the VISTA promoter had a region to which hypoxia-inducible factor (HIF)-1α was attached and resulted in increased expression of VISTA on MDSCs [100]. Kakavand et al. showed that negative immune checkpoint regulation by VISTA could be a mechanism of acquired resistance in melanoma patients treated with anti-PD-1 [42]. Moreover, adaptive resistance can be overcome by VISTA blockade. In colon cancer models, it was demonstrated that anti-VISTA therapy stimulated costimulatory genes while decreasing regulators of T-cell quiescence [101]. Although the exact correlation between VISTA and tumor resistance is not fully understood, it seems that the upregulated expression of this molecule increases resistance, and double blockade therapy with anti-PD-1 may be beneficial. Moreover, dual blockade has been shown to be advantageous when combined with chemotherapy. The expression of VISTA in tumor cells may be induced by chemotherapy via HIF-2α. The therapeutic effect of carboplatin was significantly enhanced by the implementation of the VISTA blockade [102]. The acidic microenvironment depends on cellular homeostatic mechanisms persisting mainly with transmembrane ion exchangers, including the Na^+^-H^+^ exchanger NHE1, the Na^+^-HCO−3HCO_3_-transporter NBC, and the Na^+^-dependent Cl^−^-HCO−3HCO_3_-transporter, which are acid-extruders, along with Cl^−^-HCO−3HCO_3_-exchangers [103,104,105,106]. There are few ongoing trials with these transporters as targets in gastrointestinal cancers. That is because natural bile acid receptor inhibitors (amiloride and guggulsterone) effectively suppress esophageal cancer cell growth in vitro and in nude mouse xenografts [107]. Considering this research, controlling the pH of the TME might be an option for an alternative way to modulate the therapy with VISTA inhibitors [108,109].

## 5. Clinical Trials in GITs

With the gradual emergence of specific evidence for immunotherapy resistance, combined therapy with various units and targeting different signaling pathways pose an exciting approach to investigate further [99,100,101,102,103,104]. As immunotherapy plays a pivotal role in treating various malignancies, trials of new agents and strategies are being developed as a vital part of the research. There are multiple ongoing preclinical trials aimed at VISTA, and clinical trials (NCT04475523 and NCT02812875) investigate the inhibitor agents for VISTA. The largest perspectives in preclinical studies on gastrointestinal tumors lie in targeting MDSCs and TILs, which were proven to play a key role in many malignancies. Both types of immune system cells were confirmed to be promising targets for therapy, as dysregulation within them reduces the body’s antitumor response [105,106]. Studies were performed on CT26 colon cancer in mice, where tumor CTLA-4 and PD-1 blockade failed, likely depending on the MDSC function [107]. Nevertheless, eliminating tumor granulocytic MDSCs combined with dual checkpoint blockade of PD-1 and CTLA-4 allowed the reduction of even very large tumors [107]. Blando et al. showed that anti-VISTA inhibition could restore antitumor immunological responses in pancreatic cancer by influencing an increased production of cytokines by tumor-infiltrating macrophages CD68+ [47]. The most significant study applied HMBD-002, a novel, neutralizing, anti-VISTA antibody in rats with colon cancer. The blockade resulted in a significant dose-dependent increase of IFN-γ and other inflammatory cytokines, as well as the suppression of CD14+ monocytes and tumor growth, leading to improved survival of the rats [108]. Moreover, another anti-VISTA antibody, CI-8993, has shown efficiency in trials with mice-bearing tumors and has become a substrate for clinical trials [109]. The evidence from preclinical studies clearly shows the beneficial effects of VISTA inhibitors in each advanced, metastatic, and immunotherapy-refractory tumor [110,111]. Currently, three substances are being investigated: JNJ-61610588; CI-8993; and CA-170. A few novel agents are considered for further research, with SNS-1 and W0180 being at an early stage of a clinical trial (NCT04564417; NCT05864144) [112] (Table 2).

### 5.1. JNJ-61610588

Primarily evaluated in clinical trials with positive effects, a human monoclonal antibody against VISTA has been JNJ-61610588 (NCT02671955) [113]. The 2016 phase I study included patients diagnosed with various solid tumors (pancreatic and colorectal cancer), with progression after at least one line of therapy for advanced-stage disease [26]. Twelve participants were enrolled, and the trial was divided into four parts: dose escalation; biomarker evaluation in a group of NSCLC; and two parts of dose expansion. The primary endpoints were the frequency of dose-limiting toxicity, the number of participants with adverse events (AEs), and the change from baseline in pharmacodynamic blood biomarkers. All of the stages were limited till disease progression [26]. Unfortunately, the study was terminated in 2018 due to a business decision. According to the available data, only one patient showed AE of cytokine release syndrome [112]. Despite the preterm termination of the study, efficacy has been demonstrated in individual patients. The obtained results confirmed the safety of the intravenously administered drug, with no significant side effects in most patients.

### 5.2. CI-8993

There is an ongoing phase I clinical trial (NCT04475523) assessing human immunoglobulin G1κ monoclonal antibody targeting the VISTA. The study enrolls patients with relapsed/refractory solid tumors to evaluate the safety, tolerability, and maximum tolerated dose together with an anti-cancer activity of CI-8993 in this population [114]. Primary endpoints are the determination of the maximum tolerated dose of CI-8993 and the recommended Phase II dose. Secondary endpoints are pharmacokinetic parameters of CI-8993. Since the trial was initiated in 2020, no results have been published yet. However, it seems that the high potential of blocking inactive T lymphocytes may significantly impact the TME and patients’ outcomes.

### 5.3. CA-170/AUPM-170

CA-170 is the first unique oral agent that selectively targets both VISTA and PD-L1/PD-L2. An original study on CA-170 showed antitumor efficacy in several immunocompetent mouse tumor models either as a single agent or in combination with approved therapeutics, leading to the emergence of clinical trials [115]. Sasikumar et al. described a novel mechanism of action, as CA-170 binding to PD-L1 suppressed its function but did not inhibit the formation of the PD1/PD-L1 complex, which implicated new clinical potential [115]. Phase I study (NCT02812875) enrolled patients with both lymphoma and solid tumors for whom standard therapies, including approved anti-PD-1/PD-L1, were no longer effective [116]. Inclusion criteria in this trial were, among others, tumor types known to have a high VISTA expression (such as metastatic malignant pleural mesothelioma of epithelioid histology). This study consisted of two phases with dose escalation, firstly for all the patients and later for those with PD-L1/VISTA expression. The second phase of the dose escalation aimed to explore the safety dosages and tolerability. Primary endpoints included the number of patients with dose-limiting toxicity in the first treatment cycle, along with the maximum tolerated dose of CA-170. Secondary endpoints consisted of pharmacokinetics and antitumor activity. Efficacy data from this trial was presented during the Society for Immunotherapy of Cancer (SITC) Meeting in 2018. The best-observed response was a stabilization of the disease according to the response evaluation criteria in solid tumors (RECIST). Most AEs were of grade 1–2 and included fatigue, nausea, chills, pruritus, constipation, vomiting, fever, and anorexia. AEs of grades 3–4 occurred in five patients with lipase increase, amylase increase, blood bilirubin increase, fatigue, hypokalemia, nausea, and vomiting [117]. In some cases, the treatment allowed to achieve SD for over twelve weeks and one for up to 21 weeks [118]. Considering the results of phase I clinical trial, CA-170 seems to be not only a rescue therapeutic option in patients’ refractory to previous therapy but also a completely new approach to targeting immune checkpoints. Few side effects and the possibility of oral administration facilitate the application of the therapy and may positively influence the patients’ willingness to cooperate. Phase II study of this agent was registered in India and enrolled patients with MSI-high or dMMR-positive cancers. Clinical benefit rate, which is defined by stable disease/partial response/complete response (SD/PR/CR), was observed in 52% of patients. Better results have been observed at the lower dosage, but, unfortunately, they had higher toxicities, including hypothyroidism, skin rash, and neutropenia, which could limit this treatment’s use and require further investigations [119]. The issue that requires urgent evaluation is the recurrence of neutropenia among patients who developed it as an adverse event, which eliminates a certain group of patients from treatment with this preparation [119]. Nevertheless, phase II studies seem to confirm the therapeutic success of phase I. Moreover, CA-170 is the first oral immuno-oncology agent that reached the phase II study.

## 6. Conclusions and Future Perspectives

Throughout the years, immunotherapy has emerged to be one of the most promising therapeutic options for patients with an advanced cancer diagnosis. However, in gastrointestinal tumors, a surgical approach often remains the first-line treatment, even with the occurrence of novel therapies. In light of an increasingly frequent resistance to anti-PD-1/PD-L1 and anti-CTLA-4 antibodies, there is an urgent need to investigate novel, second-generation immune checkpoint inhibitors. PD-1H, involved in several physiological responses and associated with multiple neoplasms, is currently one of the most promising post-PD-1 molecules investigated in clinical trials. However, there is still a void of knowledge in the field of precise action mechanisms, which limits the use of anti-VISTA agents [22,120]. Multiple successful trials with these agents proved them to be efficient not only as a monotherapy but also as an immunosensitizer. Targeting multiple stages of immune inhibition in cancer showed the ability to break the resistance and ensure better outcomes among patients with advanced disease [121].

Possible ways of combined immunotherapy in different tumors have been presented in many studies, including a dual blockade, as well as combining VISTA inhibitors with chemo- or radiotherapy [103,122,123]. Furthermore, we believe that it is also crucial to start combining different targets in immunotherapy. Considering the specific nature of VISTA and data from other immune checkpoint studies, a promising therapy may involve targeting angiogenesis with targeted anti-VEGF drugs and TME with immunotherapy based on anti-VISTA [124,125]. Contrarily, VISTA seems to be a multitasking agent with the potential of exertion in many more indications, thus acting, from another perspective, as an agonist [126]. As both a ligand and a receptor, VISTA presents a stronger antitumor activity than other members of the B7 family but also a wide range of possible purposes in a distinct treatment of, e.g., autoimmune diseases. Furthermore, the dissemination of knowledge about the molecule among clinical practitioners may be a problem. VISTA has demonstrated interesting but ambiguous effects in many tumors, among others, hematological neoplasia [8]. In order to establish the utility and efficacy of VISTA inhibitors amongst the possible treatment strategies of chosen cancers, further studies are required, but as for now, new mains of therapy can be opened based on this agent.

## Figures and Tables

**Table 1:** Studies on VISTA expression in gastrointestinal malignancies. ND-not detected.

Cancer Type	VISTA Expression on Tumor Cells	VISTA Expression on TMEs	Additional Data	Study	Reference
Gastric cancer	+	+	VISTA expression is correlated with Lauren type, tumor localization, etc.	Boger et al.	[41]
Gastric cancer	+	ND	Loss of VISTA expression is a recurrent event correlated with the microenvironment of the tumor.	Wang et al.	[51]
Hepatocellular carcinoma	+	ND	Dual positive tumors (VISTA +/CD8+) showed better OS.	Zhang et al.	[48]
Hepatocellular carcinoma	+	ND	Blocking VISTA can suppress tumor growth.	Lei et al.	[8]
Colorectal carcinoma	+	+	VISTA was mainly expressed on tumor infiltrating leukocytes; expression was higher than PD-L1.	Xie et al.	[28]
Esophageal adenocarcinoma	+	+	Favorable outcome of VISTA-positive tumors.	Loeser et al.	[52,53]
Pancreatic ductal adenocarcinoma	ND	+	Higher density of VISTA expression on CD38+ macrophages in contrast to melanoma.	Blando et al.	[49]

**Table 2:** Ongoing clinical trials with VISTA inhibitors.

Medication	Target	Population	Status	Phase	NCT
JNJ-61610588	VISTA	Advanced solid tumors progressed after prior treatment.	Terminated	I	NCT02671955
CI-8993	VISTA	Relapse/refractory solid tumors.	Active, not recruiting	I	NCT04475523
CA-170	VISTA PD-L1	Advanced solid tumors or lymphomas	Completed	I	NCT02812875
	PD-L2	Lung cancer, head/neck/oral cavity cancer, MSI-H positive cancers, and Hodgkin lymphoma	Terminated	II	CTRI/2017/12/011026 (India)
W0180	VISTA	Histologically confirmed solid tumors progressed after standard treatment	Recruiting	I	NCT04564417
SNS-1	VISTA	Histologically or cytologically documented locally advanced, unresectable, or metastatic solid tumors.	Recruiting	I/II	NCT05864144

## Data Availability

Not applicable.

## Abbreviations

AE	adverse event
APC	antigen presenting cell
CR	complete response
CTLA-4	cytotoxic T cell antigen 4
ECD	extracellular domain
GIT	gastrointestinal tumor
dMMR	mismatch repair deficiency
MDSC	myeloid-derived suppressor cells
MSI-H	microsatellite instability
OS	overall survival
PD-1/PD-L1	programmed death receptor 1/programmed cell death ligand 1
PR	partial response
PSGL-1	P-selectin glycoprotein ligand-1
RR	response rate
RECIST	response evaluation criteria in solid tumors
SD	stable disease
TIL	tumor infiltrating lymphocytes
VISTA	V-domain Ig suppressor of T-cell activation
VSIG3	V-set and immunoglobulin domain containing 3
VSIR	V-Set Immunoregulatory Receptor
